# Splice rescue of protein-truncating variants is associated with reduced selection pressure and attenuated disease phenotypes

**DOI:** 10.1016/j.gendis.2026.102093

**Published:** 2026-02-14

**Authors:** Xi Cheng, Fei Liu, Yixuan Gai, Lina Zhao, Wen Wen, Xueqing Lu, Zhihong Wu, Jianguo Zhang, Sen Zhao, Nan Wu

**Affiliations:** a Department of Orthopedic Surgery, Peking Union Medical College Hospital, Peking Union Medical College and Chinese Academy of Medical Sciences, Beijing 100730, China; b Department of Dermatology, The First Affiliated Hospital, Zhejiang University School of Medicine, Hangzhou, Zhejiang 310000, China; c State Key Laboratory of Complex Severe and Rare Diseases, Peking Union Medical College Hospital, Peking Union Medical College and Chinese Academy of Medical Sciences, Beijing 100730, China; d Beijing Key Laboratory for Genetic Research of Skeletal Deformity, Beijing 100730, China; e Stem Cell Facility, Institute of Clinical Medicine, Peking Union Medical College Hospital, Chinese Academy of Medical Sciences, Beijing 100730, China; f Key Laboratory of Big Data for Spinal Deformities, Chinese Academy of Medical Sciences, Beijing 100730, China

The deleterious effect of putative loss-of-function (LoF) variants could be rescued by various genetic compensation mechanisms, such as in-frame exon skipping/intron retention, the absence of the variant in impactful transcripts, or a second variant on the same haplotype that restores the reading frame. These events are often overlooked by variant annotation tools, presenting challenges in variant interpretation. Here we report a novel splice rescue mechanism where protein-truncating variants (PTVs, *i.e.*, nonsense variants and frameshift variants) introduce a cryptic splice site, leading to an in-frame splice-out and consequent preservation of protein function (Fig. 1A and B). To our knowledge, the rescue mechanism has neither been acknowledged in variant interpretation guidelines nor systematically explored.

**Figure 1:**
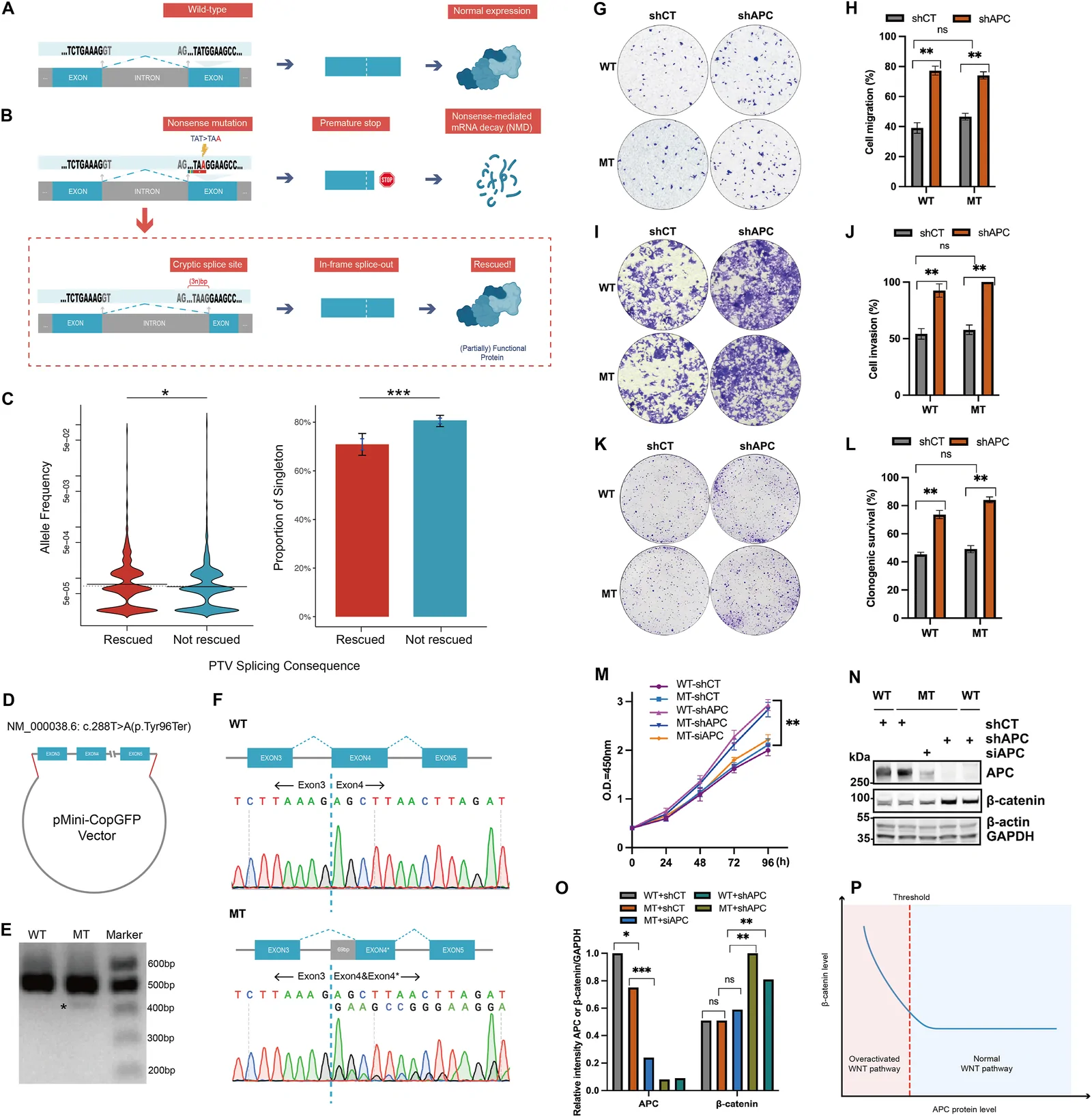
Splice rescue mechanism and verification on *APC* nonsense variant. **(A)** Normal transcription and expression process without protein-truncating variant (PTV). **(B)** Canonical transcription outcome of a PTV (upper part); Splice rescue where the activation of a cryptic splice site leads to an in-frame splice-out of this variant, resulting in a partially functional protein (lower panel in a red dashed box). **(C)** Allele frequency (left panel) and proportion of singletons (right panel) between in-frame and out-of-frame PTVs. ∗*P* < 0.05 and ∗∗∗*P* < 0.001. **(D)** Minigene construction with the *APC* nonsense variant. **(E)** Gel electrophoresis of reverse transcription-PCR products of wild-type (WT) and mutant-type (MT) plasmids. The asterisk (∗) indicates the rescued transcript. **(F)** Sanger sequencing of reverse transcription-PCR products showing *APC* exon 3-5 of WT and MT plasmids. **(G**–**L)** Typical images and efficiencies of cell migration (G, H), cell invasion (I, J), and colony formation (K, L) in *APC* WT/MT cell ± shAPC/shCT. **(M)** Growth curves of *APC* WT/MT cell ± shAPC/shCT, and *APC* MT cell + siAPC. **(N)** Western blotting assay detected the correlation between APC protein and β-catenin. **(O)** Quantification of the protein level of APC or β-catenin. ∗*P* < 0.05, ∗∗*P* < 0.01, and ∗∗∗*P* < 0.001; ns = not statistically significant. **(P)** Threshold effect between APC protein level and β-catenin level. The red dashed line represents the threshold level of APC protein. The blue shaded area indicates a normal β-catenin level, maintaining proper WNT pathway function, while the pink shaded area represents high β-catenin levels, leading to overactivation of the WNT pathway.

To preliminarily explore the prevalence of splice rescue, we screened all PTVs (*n* = 346,433) in the gnomAD database (v2.1.1) using SpliceAI,^1^ with splicing predictions restricted to MANE Select transcripts (Fig. S1). The selection criteria are: i) the length of the predicted spliced-out sequence was a multiple of three; ii) the PTV is located within the spliced-out sequence. We found 10,071 (2.9%) of PTVs predicted to be splice-altering. Among these splice-altering PTVs, 1035 (10.3%) would generate spliced-out transcripts predicted to be in-frame. In summary, PTVs associated with splice rescue respectively account for 0.3% of all PTVs and 10.3% of PTVs predicted to affect splicing. From the evolutionary perspective, PTVs are generally subject to strong purifying selection, resulting in extremely low allele frequencies and a high proportion of singletons in the population. We found that PTVs associated with splice rescue exhibited higher allele frequencies (*P* = 0.043) and a lower proportion of singletons (*P* < 0.001) compared with those that did not undergo splice rescue (Fig. 1C), while no significant differences were found in missense variants (Fig. S2). This observation suggests that these PTVs associated with splice rescue might be subject to reduced selection pressure owing to less harmful consequences.

Although uncommon, splice rescue is hypothesized to explain some of the phenotypic variability observed in certain diseases, challenging the deleterious classification of certain known “pathogenic” variants. To further validate splice rescue events, we retrieved all PTVs from the ClinVar database and analyzed the splicing consequences of these variants. Among 115,959 PTVs, 237 (0.2%) variants were predicted to undergo splice rescue, 209 of which were classified as “pathogenic” or “likely pathogenic” (Table S1). Here, we highlighted a nonsense variant in the *APC* gene associated with the attenuated familial adenomatous polyposis (AFAP), to demonstrate the genotype-phenotype correlation underlying the splice rescue mechanism.

AFAP is a milder form of the classic familial adenomatous polyposis (FAP). Patients with AFAP typically present fewer than 100 polyps, later-onset colorectal cancer, and less extra-intestinal manifestation.^2^ An *APC* nonsense variant (NM_000038.6: c.288T>A, p.Tyr96Ter), which was classified as “pathogenic” by ClinVar, was reported to cause AFAP. We predicted that this variant would undergo splice rescue with a 69-bp sequence splice-out, including the variant itself. A minigene assay confirmed the splicing products with a 69-bp deletion with gel electrophoresis and Sanger sequencing, though in a small amount (Fig. 1D–F). Intriguingly, another nonsense variant at the same amino acid (NM_000038.6: c.288T>G) but without splice rescue was reported to be associated with classic FAP.^3^ Mutations at the same nucleotide position lead to different disease outcomes, suggesting that the splice rescue mechanism might explain the milder disease phenotypes observed in patients harboring the c.288T>A variant. To further confirm the generalizability of the splice rescue mechanism, we identified and experimentally validated another predicted event in *QRICH2* (NM_032134.2: c.3037C>T [p.Arg1013Ter]), which was predicted to produce a 183-bp in-frame splice-out. A minigene splicing assay confirmed the presence of the rescued transcript (Supplementary results; Fig. S4).

To further understand the biological effects of the *APC* variant, we constructed an *APC* mutant cell line with a point mutation (c.288T>A) in the HCT116 cell line and introduced stable expression of anti-human *APC* shRNA (shAPC) or control shRNA (shCT) in both the wild-type and mutant cell lines. Results showed that the *APC* mutant cell line (MT + shCT) exhibited comparable biological function as the wild-type cell line (WT + shCT) across several key cellular processes, including cell migration (Fig. 1G and H), cell invasion (Fig. 1I and J), colony formation (Fig. 1K and L), and cell growth (Fig. 1M). The similarity indicates that the effect of this *APC* mutation is milder than a complete loss of function, underscoring its less deleterious nature.

The APC protein plays a critical role in the canonical WNT signaling pathway by inhibiting β-catenin activity. Next, we aimed to examine alterations in downstream β-catenin levels in the *APC* mutant cell line. To investigate the dose–response relationship between APC protein and β-catenin, we employed anti-*APC* small interfering RNA (siAPC) to transiently inhibit *APC* expression. In the stable *APC* knockdown cell lines (MT + shAPC, WT + shAPC), minimal *APC* expression resulted in elevated β-catenin expression. In the absence of shAPC interference, the *APC* mutant (MT + shCT) cell line displayed high *APC* expression, though lower than that in the wild-type cell line (WT + shCT). In the *APC* mutant cell line treated with siAPC (MT + siAPC), *APC* expression was intermediate between MT + shCT (*APC* expression not inhibited) and MT + shAPC (*APC* expression consistently inhibited) as expected, though significantly lower than the expression in MT + shCT (Fig. 1N and O). Interestingly, despite significant differences in *APC* expression among the WT + shCT, MT + shCT, and MT + siAPC groups (*i.e.*, *APC* expression not fully inhibited groups), β-catenin levels were consistent (Fig. 1O), and their growth curves were similar as well (Fig. 1M). The results indicate that partial loss of APC protein might be tolerable, almost without adverse effects on cellular processes. Therefore, the correlation between APC protein and β-catenin in HCT116 cells may not be inversely proportional but subject to a threshold effect (Fig. 1P). To further validate this hypothesis, we performed a dose–response experiment using increasing concentrations of siAPC (0, 5, 10, 20, and 30 nM) and measured the corresponding β-catenin levels. Sigmoid curve fitting revealed a clear threshold-like relationship, supporting the proposed non-linear correlation between APC reduction and β-catenin accumulation (Fig. S5). This finding further deepened our understanding of the variant's role in causing AFAP: in the early stages, rescued APC protein levels above a certain threshold can maintain the normal biological function of the WNT pathway. However, as APC protein deficiency expands due to loss of heterozygosity or mutation accumulation, APC protein levels drop below this threshold and are not sufficient to execute appropriate biological functions, leading to later-onset tumorigenesis. The threshold effect was previously demonstrated in the mouse model, estimating the threshold at approximately 15% of the wild-type protein level.^4^

Deciphering the role of the *APC* variant in AFAP inspired two novel therapeutic approaches for this disease: enhancing *APC* rescue transcript expression via antisense oligonucleotides (ASOs) and inhibiting downstream function in the WNT signaling pathway. Through cellular verification, we observed that ASO significantly increased rescued transcript levels by blocking canonical splicing (*i.e.*, the constitutive splicing process occurring at consensus splice junctions), thereby enhancing the rescue effect (Fig. S3). To quantitatively evaluate this effect, we performed reverse transcription-PCR followed by PSI (Percent Spliced In) analysis, which revealed that the rescued isoform accounted for 92.5% in the MT-ASO group and 100% in the MT+ASO group (Fig. S3). In light of this, a genetic therapy that leverages both ASO and rescue mechanisms emerges as a promising avenue for future treatments.

Our results highlight the clinical importance of considering splice rescue in variant interpretation. According to the current ACMG/AMP framework, protein-truncating variants are often assigned the PVS1 criterion (“null variant … in a gene where loss of function is a known mechanism of disease”).^5^ However, when such a variant undergoes in-frame splice rescue and retains partial protein function, the application of *PVS1* at a “very strong” level may overestimate pathogenicity. Consistent with recent proposals to calibrate *PVS1* strength for variants escaping NMD or producing residual activity, we suggest that verified or computationally predicted splice rescue events should warrant downgrading *PVS1* to a lower level of evidence. Incorporating splice rescue into variant assessment frameworks would refine pathogenicity classification, mitigate potential overdiagnosis, and enhance the accuracy of molecular diagnosis—particularly for variants associated with attenuated phenotypes. At the same time, we also acknowledge that population structure and annotation differences in gnomAD may influence the estimated prevalence of splice rescue events, while future ancestry-stratified datasets will enable more refined assessments.

In conclusion, our study delineates a splice rescue mechanism associated with reduced selection pressure and attenuated disease phenotype. Although rare, the strategy to escape negative selection might elucidate why some patients exhibit mild phenotypes despite carrying “pathogenic” variants. Splice rescue variants warrant careful interpretation, particularly in avoiding overdiagnosis in patients with mild clinical presentations. This understanding furnishes novel insights for refining variant interpretation in both research and clinical settings, identifying potential therapeutic targets, and investigating the complex interplay between human genetics and disease.

## CRediT authorship contribution statement

**Xi Cheng:** Writing – original draft, Formal analysis, Data curation, Conceptualization. **Fei Liu:** Writing – original draft, Formal analysis, Data curation, Conceptualization. **Yixuan Gai:** Investigation, Formal analysis, Data curation. **Lina Zhao:** Methodology, Investigation, Formal analysis. **Wen Wen:** Investigation, Data curation. **Xueqing Lu:** Resources, Methodology, Formal analysis. **Zhihong Wu:** Validation, Supervision, Funding acquisition. **Jianguo Zhang:** Supervision, Funding acquisition. **Sen Zhao:** Writing – review & editing, Supervision. **Nan Wu:** Writing – review & editing, Supervision, Funding acquisition.

## Ethics declaration

The study does not involve human participants, individual-level data collected from humans, or bio-samples collected from humans.

## Data availability

The data that support the findings of this study are available on request from the corresponding author, Nan Wu (dr.wunan@pumch.cn).

## Funding

This research was funded in part by the 10.13039/501100001809National Natural Science Foundation of China (No. 82572698, 82172382 to J.Z.), the 10.13039/501100012166National Key Research and Development Program of China (No. 2022YFC2703102 to N.W., 2022YFC2703901 to Z.W.), the National High Level Hospital Clinical Research Funding (China) (No. 2022-PUMCH-D-004 to J.Z. and N.W., 2022-PUMCH-D-002 to Z.W.), CAMS Innovation Fund for Medical Sciences (No. 2024-I2M-TS-002 to N.W.; 2021-I2M-1-051 to J.Z. and N.W.; 2021-I2M-1-052, 2022-I2M-2-001 to Z.W.; 2023-I2M-C&T-A-003 to J.Z.), Peking Union Medical College Hospital Talent Cultivation Program (Category A) (No. ULJ11240) to N.W. and the National Key Clinical Specialty Construction Project and the Non-profit Central Research Institute Fund of Chinese Academy of Medical Sciences (No. 2019PT320025).

## Conflict of interests

The authors declared no competing interests.
