# Supplementary material for: Splice rescue of protein-truncating variants is associated with reduced selection pressure and attenuated disease phenotypes
Source: Genes Dis. 2026 Feb 14;14(1):102093. doi: 10.1016/j.gendis.2026.102093 (PMC13545357; doi:10.1016/j.gendis.2026.102093)
Supplement: Supplementary file 1 [file mmc1.docx]

**Splice rescue of protein-truncating variants is associated with reduced selection pressure and attenuated disease phenotypes**

Xi Cheng, Fei Liu, Yixuan Gai, Lina Zhao, Wen Wen, Xueqing Lu, Zhihong Wu, Jianguo Zhang, Sen Zhao, Nan Wu

**Contents**

Supplementary Methods

Supplementary Results

Supplementary Figures 1-5

References

**Supplementary Methods**

***Data collection***

The Genome Aggregation Database, gnomAD v2.1.1(1) dataset (<https://gnomad.broadinstitute.org/>, accession date: 2022-6-8), which consisted of 125,748 exome sequencing (ES) and 15,708 genome sequencing (GS) data, was used to retrieve known human coding variants and their populational frequencies. Only those variants that passed the gnomAD build-in hard filters were included. We also collected nonsense and frameshift variants associated with Mendelian diseases from the ClinVar(2) dataset (https://www.ncbi.nlm.nih.gov/clinvar/, accession date: 2022-04-25, hg19). Variants were manually curated independently by two scientists according to the American College of Medical Genetics and Genomics/Association for Molecular Pathology (ACMG/AMP) guideline(3).

***Data Annotation***

We used Ensembl VEP (Variant Effect Predictor)(4) software to annotate variants. SpliceAI plugin (https://github.com/Illumina/SpliceAI/)(5) was integrated into VEP for predicting splicing sites and probabilities. SpliceAI is a convolutional neural network-based algorithm for identifying splicing variants for all SNVs and small indels. It provided specific delta scores (here we used a recommended threshold of 0.5) and delta positions for donor gain (DG), donor loss (AL), acceptor gain (AG), and acceptor loss (AL). For each variant, we chose delta scores of the MANE Selected transcript(6), which best represented the biology of the gene.

***Data selection and calculation***

The exon-intron boundaries, which locate canonical splicing sites (GT/AG at the end of nearby introns), were downloaded in BED files from UCSC Table Browser (<http://genome.ucsc.edu/cgi-bin/hgTables>, CCDS track, hg19). The delta position of SpliceAI helped identify the location of the predicted splicing site, and thus the base number of the spliced-out sequence could be calculated by subtracting the predicted splicing site from the canonical splicing site. Notably, the variant itself should be included within the spliced-out sequence. If the length of the predicted spliced-out sequence was a multiple of three, the splicing consequence was defined as ‘in-frame splicing’ and thus the possible deleterious effect caused by the variant was assumed to be ‘rescued’. Vice versa, if the length was not a multiple of three, the splicing consequence was defined as ‘out-of-frame splicing’ and the possible deleterious effect caused by the variant was assumed to be ‘not-rescued’. Here we focused on the evaluation of the consequences when the delta score of AG or DG > 0.5. However, we did not consider the effects of solely AL or DL > 0.5, as the new location of the splice site could not be inferred (defined as ‘ambiguous’). The consequences of both AL and DL > 0.5, which correspond to the result of exon skipping, were also calculated. The workflow of data collection, annotation, selection, and calculation are integrated into Supplementary Figure 1.

***Minigene assay***

For candidate variants NM_000038.6:c.288T>A locating at exon 4 of *APC* gene, a minigene assay was used to evaluate the potential splicing consequence. The region of the APC gene spanning exons 3 to 5 covers more than 9 kilobases, with intron 4 comprising nearly 8 kilobases. Given that 200 base pairs of the intronic regions flanking the exons are sufficient for accurate mRNA splicing (7, 8), we designed a minigene that includes exon 3, intron 3, exon 4, the first 300 base pairs of intron 4, the last 300 base pairs of intron 4, and exon 5.The minigene regions of the *APC* gene were cloned into the pMini-CopGFP vector (Beijing Hitrobio Biotechnology Co., Ltd.), validated by Sanger sequencing. The recombinant plasmids were transfected into HEK293T cells with Lipofectamine 2000 (Invitrogen) when the cell confluency grew to 50% to 60%. After incubation at 37℃, 5% CO_2_ for 48h, total RNA was extracted from cells by the RNeasy Mini Kit (Qiagen, Germany) according to the standard protocol. Reverse transcription of purified RNA was conducted by the PrimeScript RT reagent kit (Takara, Japan). PCR fragment products were analyzed by agarose gel electrophoresis and Sanger sequencing to detect abnormal splicing products. Sequencing results were analyzed *in silico* by SnapGene software (version 4.3.6).

Primer for canonical splicing transcript:

Forward: 5’-GCGTCTTAAAGAGCTTAACTT-3’

Reverse: 5’-AAGCAATGACCTCTCTTTCT-3’

Primer for rescue transcript:

Forward: 5’-CTTAAAGGAAGCCGGGAAG-3’

Reverse: 5’-AGCAATGACCTCTCTTTCTCAA-3’

***Cell line construction, culture, and transfection***

Wild-type HCT116 (WT) and homozygous *APC* mutant (NM_000038.6:c.288T>A) HCT116 (MT) cell lines were purchased from Ubigene (<https://www.ubigene.com>). ASO specifically targeting *APC* (ASO-APC) was designed by IDT (Integrated DNA Technologies, Inc.) with 2’-MOE (phosphorothioate and 2′-O-methoxyethyl) modification and standard desalting purification, and a general negative control ASO-CT was designed as Gene Tools, LLC (<https://www.gene-tools.com/content/negative-control-morpholino-oligos>). The specific shRNA targeting *APC* (shAPC) and the non-targeting control shRNA (shCT) were synthesized by Ubigene. The specific siRNA targeting *APC* (siAPC) and the non-targeting control siRNA (siCT) were synthesized by GenePharma.

The sequence of ASOs, siRNA, and shRNAs are provided as below:

ASO (targeting *APC*): /52MOErG/*/i2MOErC/*/i2MOErT/*/i2MOErA/*/i2MOErT/*/i2MOErC/*/i2MOErT/*/i2MOErA/*/i2MOErA/*/i2MOErG/*/i2MOErT/*/i2MOErT/*/i2MOErA/*/i2MOErA/*/i2MOErG/*/i2MOErC/*/i2MOErT/*/i2MOErC/*/i2MOErT/*/i2MOErA/*/i2MOErA/*/32MOErA/

ASO (negative control):

/52MOErC/*/i2MOErC/*/i2MOErT/*/i2MOErC/*/i2MOErT/*/i2MOErT/*/i2MOErA/*/i2MOErC/*/i2MOErC/*/i2MOErT/*/i2MOErC/*/i2MOErA/*/i2MOErG/*/i2MOErT/*/i2MOErT/*/i2MOErA/*/i2MOErC/*/i2MOErA/*/i2MOErA/*/i2MOErT/*/i2MOErT/*/i2MOErT/*/i2MOErA/*/i2MOErT/*/32MOErA/

(Note: *, phosphorothioate; 2MOE, 2’-O-methoxyethyl.)

siAPC: TCTATTTCAGGAACCAAACAAAGTA

shAPC: AGTACAAGGATGCCAATATTA

shCT: CCTAAGGTTAAGTCGCCCTCG

For transfection, cells were transfected with siRNAs/ASO using Jet PRIME kits (Polyplus, 101000046) following the manufacturer's protocol. WT/MT cells were infected with the above Lentivirus expressing shAPC/shCT with 10ug/ml polybrene, screened with puromycin for resistance, and obtained *APC* knock-down and control stable cell lines (WT + shAPC, MT + shAPC, WT + shCT, MT +shCT).

***APC*** ***Cell invasion and migration assay***

For the Transwell invasion assay, approximately 1×10^5^ cells were added to the upper chamber (Corning, 3422) coated with Matrigel in 100μL of serum-free McCoy’s 5A medium, and 600μL of McCoy’s 5A medium with 10% FBS was added to the lower chamber. After 48h, the cells remaining inside of the upper chamber were removed with a wet cotton swab, and the chambers were fixed with 4% paraformaldehyde and then stained with 0.1% crystal violet. The invading cells were counted in five randomly selected microscopic views. For the Transwell migration assay, a procedure similar to the Transwell invasion assay was performed using the same migration chamber without Matrigel. All experiments were performed in triplicate.

***APC Cell proliferation assay and colony formation assay***

For the cell proliferation assay, *APC* stable knock-down cell lines WT + shAPC or MT + shAPC and control stable knock-down cell lines WT + CT or MT + CT were plated at a density of 2000 cells per well in 96-well plates and cultured for 4 days. A total of 10μL of CCK-8 reagent (KeyGEN, China) was added daily, at the same time, to each well and incubated for 1h. Cell viability was measured according to the optical density at 450 nm. For the colony formation assay, *APC* stable knock-down cell lines WT + shAPC or MT + shAPC and control stable knock-down cell lines WT + CT or MT + CT were seeded in 6-well plates at a density of 2000 cells per well and cultured for about two weeks. After methanol fixation and staining with 0.1% crystal violet, cell colonies were counted. All experiments were performed in triplicate.

***Western blot analysis***

*APC* stable knock-down WT + shAPC or MT + shAPC cells and control stable knock-down WT + CT or MT + CT cells were grown to 90% confluency in 6-well plates were washed with cold phosphate-buffered saline (PBS), then the cells were lysed with 100µL Western Cell Lysis buffer (Beyotime, P0013) containing protease inhibitor cocktail (Sigma, P8340). Total proteins were separated by SDS-PAGE and transferred to PVDF membranes (Millipore, IPVH00010). The membranes were blocked with 5% skim milk at room temperature for 1h, then incubated with primary antibodies overnight at 4°C. The primary antibodies include APC (abcam, ab40778), β-catenin (abcam, ab32572), β-actin (abcam, ab8226), and GAPDH (Immunoway, YM3029). After washing three times with PBS containing 0.1% Tween-20, the membranes were incubated with secondary antibodies (Cell Signaling Technology, USA) at room temperature for 1h. Proteins were visualized using ChemiDoc™ Imaging System (Bio-RAD, 12003153). The grayscale scanning software ImageJ software (NIH, Bethesda, MD) was used for Western blot images to quantify the relative expression of the target proteins. All experiments were performed in triplicate.

***Statistics***

Statistical analysis was performed with Python (version 3.8.3) and R (Version 4.0.3) for calculation and visualization. For public datasets, data were analyzed by the Wilcoxon test (for allele frequency) and the Chi-Square test (for proportion of singleton), in which a *p*-value of less than 0.05 in a one-sided test was considered statistically significant. For molecular experiments, statistical analysis and visualization were conducted using GraphPad Prism 10 software (GraphPad Software, USA), and the significance was determined using a 2-tailed, unpaired, Student *t*-test or 2-way ANOVA analysis with Bonferroni post hoc test.

**Supplementary Results**

***Splice Rescue of*** ***QRICH2 variant***

From the ClinVar database, we identified a nonsense variant in the *QRICH2* gene (NM_032134.2: c.3037C>T[p.Arg1013Ter]) that was predicted by SpliceAI to result in a 183bp sequence splice-out, including the nonsense variant itself. Based on the ACMG/AMP guideline, this variant was classified as ‘pathogenic’ (PVS1+PM2+PP3+PP5). It was interpreted by the ClinVar database as ‘pathogenic’ as well, albeit with a zero-star review status (no assertion criteria provided). As reported previously by Shen et al. (9), the homozygous form of this variant was shown to decrease gene expression, leading to multiple morphological abnormalities of the flagella (MMAF). Another *QRICH2* nonsense variant (NM_032134.2: c.192G>A[p.Leu64Ter]) was also identified as pathogenic in their research. Interestingly, their research revealed a small amount of sperm motility (5.4%) and progressive motility (2.7%) in the patient homozygous for the c.3037C>T variant, whereas both sperm motility and progressive motility metrics were zero in the patient homozygous for the c.192G>A variant (**refer to Shen et al. Table 1**). Similarly, immunofluorescence staining on human spermatozoa showed slight expression of QRICH2 protein in the sperm of c.3037C>T homozygous patient, but a complete absence in that of c.192G>A homozygous patient (**refer to Shen et al. Figure 2**). The evidence suggests that the c.3037C>T variant may slightly retain sperm activity, which is potentially attributed to cryptic splice site activation and splicing rescue. To validate the abnormal splicing predicted by SpliceAI, we conducted a minigene assay for the c.3037C>T variant. The results of agarose gel electrophoresis and Sanger sequencing confirmed the presence of the transcript with a 183bp deletion, further supporting the splicing rescue hypothesis (**Supplementary Figure 4**).

**Supplementary figures**

**Supplementary Figure 1. Workflow for defining potentially rescued variants.** This flowchart outlines the step-by-step process used to select and define potentially rescued variants from the gnomAD and ClinVar database, including quality control, annotation, selection, and calculation. Variants were then categorized based on their predicted splicing status into potentially ‘rescued’ and ‘not-rescued’ variants. SpliceAI_pred_DS_AG, delta score for acceptor gain; SpliceAI_pred_DS_DG, delta score for donor gain.

**Supplementary Figure 2. Allele frequency and proportion of singleton in gnomAD missense variants associated with alternative splicing.** Allele frequency (left panel) and proportion of singletons (right panel) between in-frame and out-of-frame missense variants. ns = not statistically significant.

**Supplementary Figure 3. Inhibition of canonical transcription by ASO promotes splice rescue in the *APC* mutant cell line**. A. The levels of canonical transcript and rescue transcript in *APC* mutant-type (MT) HCT116 cell lines with or without antisense oligonucleotide (ASO). B. qPCR detected the relative expression of canonical transcript (Exon3+Exon4) and rescue transcript (Exon3+△Exon4) in *APC* wild-type (WT) or MT cell lines with or without ASO. ***P<0.001. C. RT-PCR and agarose gel electrophoresis confirming the presence of canonical and rescued transcripts in WT and MT cells with or without ASO treatment. The percent spliced-in (PSI) values were calculated from band intensities. The asterisk indicates a faint band of canonical transcript.

**Supplementary Figure 4. Splice rescue of *QRICH2* nonsense variant and minigene assay validation.** A. Schematic representation contrasting the predicted splice rescue mechanism for a *QRICH2* nonsense variant, where an in-frame splice-out of the variant leads to the production of a partially functional protein (highlighted in the lower panel in a red dashed box), against the canonical splicing pathway (depicted in the upper panel with a grey shade). The splice rescue of the variant corresponds to low sperm motility. B. Minigene construction designed to include the *QRICH2* nonsense variant. C. Sanger sequencing results showcasing *QRICH2* exon 4 to exon 5 in wild-type (WT) and mutant-type (MT) cells derived from the minigene assay. The MT results validate the existence of rescue transcripts.

**Supplementary Figure 5. Dose-dependent relationship between APC suppression and β-catenin accumulation.** A. Western blot analysis of APC and β-catenin protein levels in MT (c.288T>A) cells transfected with increasing concentrations of APC-targeting siRNA (0, 5, 10, 20, and 30 nM). shAPC was included as a complete knockdown control. β-actin served as the loading control. The relative intensity of β-catenin normalized to β-actin is indicated below each lane. B. Sigmoid fitting curve illustrating the relationship between siAPC concentration and relative β-catenin levels from the Western blot. The shaded regions denote the normal (blue) and overactivated (pink) WNT pathway states.

**References**

1.	Karczewski KJ, Francioli LC, Tiao G, Cummings BB, Alföldi J, Wang Q, et al. The mutational constraint spectrum quantified from variation in 141,456 humans. Nature. 2020;581(7809):434-43.

2.	Landrum MJ, Lee JM, Benson M, Brown GR, Chao C, Chitipiralla S, et al. ClinVar: improving access to variant interpretations and supporting evidence. Nucleic Acids Res. 2018;46(D1):D1062-d7.

3.	Richards S, Aziz N, Bale S, Bick D, Das S, Gastier-Foster J, et al. Standards and guidelines for the interpretation of sequence variants: a joint consensus recommendation of the American College of Medical Genetics and Genomics and the Association for Molecular Pathology. Genet Med. 2015;17(5):405-24.

4.	McLaren W, Gil L, Hunt SE, Riat HS, Ritchie GRS, Thormann A, et al. The Ensembl Variant Effect Predictor. Genome Biology. 2016;17(1):122.

5.	Jaganathan K, Kyriazopoulou Panagiotopoulou S, McRae JF, Darbandi SF, Knowles D, Li YI, et al. Predicting Splicing from Primary Sequence with Deep Learning. Cell. 2019;176(3):535-48.e24.

6.	Cunningham F, Allen JE, Allen J, Alvarez-Jarreta J, Amode MR, Armean IM, et al. Ensembl 2022. Nucleic acids research. 2022;50(D1):D988-D95.

7.	Becirovic E, Böhm S, Nguyen ONP, Riedmayr LM, Koch MA, Schulze E, et al. In vivo analysis of disease-associated point mutations unveils profound differences in mRNA splicing of peripherin-2 in rod and cone photoreceptors. PLoS genetics. 2016;12(1):e1005811.

8.	Nguyen OP, Böhm S, Gießl A, Butz ES, Wolfrum U, Brandstätter JH, et al. Peripherin-2 differentially interacts with cone opsins in outer segments of cone photoreceptors. Human molecular genetics. 2016;25(12):2367-77.

9.	Shen Y, Zhang F, Li F, Jiang X, Yang Y, Li X, et al. Loss-of-function mutations in QRICH2 cause male infertility with multiple morphological abnormalities of the sperm flagella. Nature communications. 2019;10(1):1-15.
